# Conflict adaptation and related neuronal processing in Parkinson’s disease

**DOI:** 10.1007/s11682-021-00520-w

**Published:** 2021-08-27

**Authors:** Rea Rodriguez-Raecke, Christoph Schrader, Pawel Tacik, Dirk Dressler, Heinrich Lanfermann, Matthias Wittfoth

**Affiliations:** 1 Diagnostic and Interventional Neuroradiology, University Hospital, RWTH Aachen University, Pauwelsstraße 30, 52074 Aachen, Germany; 2 Department of Neurology, Hannover Medical School, Hannover, Germany; 3 Department of Neuroradiology, Hannover Medical School, Hannover, Germany; 4 Department of Neurodegenerative Diseases and Geriatric Psychiatry, University of Bonn Medical Center, Bonn, Germany

**Keywords:** fMRI, Congruency sequence effect, Parkinson’s disease, Cognitive control, Conflict adaptation

## Abstract

Non-motor symptoms like cognitive impairment are a huge burden for patients with Parkinson’s disease. We examined conflict adaptation by using the congruency sequence effect as an index of adaptation in 17 patients with Parkinson’s disease and 18 healthy controls with an Eriksen flanker task using functional magnet resonance imaging to reveal possible differences in executive function performance. We observed overall increased response times in patients with Parkinson’s disease compared to healthy controls. A flanker interference effect and congruency sequence effect occurred in both groups. A significant interaction of current and previous trial type was revealed, but no effect of response sequence concerning left or right motor responses. Therefore, top-down conflict monitoring processes are likely the main contributors leading to the congruency sequence effect in our paradigm. In both groups incongruent flanker events elicited activation in the middle temporal gyrus, inferior parietal cortex, dorsolateral prefrontal cortex and the insula in contrast to congruent flanker events. A psychophysiological interactions analysis revealed increased functional connectivity of inferior parietal cortex as a seed to the left prefrontal thalamus during incongruent vs. congruent and neutral stimuli in patients with Parkinson’s disease that may reflect compensatory facilitating action selection processes. We conclude that patients with Parkinson’s disease exhibit conflict adaptation comparable to healthy controls when investigated while receiving their usual medication.

**Supplementary Information:**

The online version contains supplementary material available at 10.1007/s11682-021-00520-w.

## Introduction

Parkinson's disease (PD) is the second most common neurodegenerative disorder worldwide and is marked by motor dysfunctions as well as non-motor symptoms (Schapira et al., 2017). Conflict processing tasks like the flanker task (Eriksen & Eriksen, 1974) are used to investigate cognitive control and inhibitory action control (Ridderinkhof et al., 2011). In healthy controls (HC), response times (RT) following congruent flanker events (C) are shorter compared to incongruent flanker events (IC), which is known as congruency effect. In patients with PD, several studies showed further slowing of RTs for incongruent flanker events (Claassen & Wylie, 2012; Wylie et al., 2005) and larger congruency effects (Praamstra et al., 1998, 1999). Not consistent with these findings, other studies could not reveal an emphasized congruency effect in PD (Falkenstein et al., 2006). This might be connected to differing target onset delays in the task (Cagigas et al., 2007). Further, congruency effects are also reported to be linked to medication status in PD (Djamshidian et al., 2011). In PD, facilitation effects, referring to shorter RTs following congruent flanker events compared to neutral flanker events (N), are not reported to show significant differences to HC (Falkenstein et al., 2006; Wylie et al., 2005). Investigating RTs as a function of specific sequences of flanker events, the congruency effect is reportedly reduced if a specific trial follows directly after incongruent as compared to congruent trials. This effect is referred to as “congruency sequence effect (CSE)” (Duthoo et al., 2014). There are several approaches to explain this effect and to evaluate how much of it is actually a top-down process with attentional control continuously monitoring the processing stream (Botvinick et al., 2001), or a bottom-up episodic memory effect mimicking the congruency sequence effect (Hommel et al., 2004; Mayr et al., 2003). The conflict monitoring theory describes conflict adaptation as shorter RTs for incongruent trials following other incongruent trials. Further, the concept of conflict adaptation states that a similar level of conflict during consecutive flanker events reduces RTs (Botvinick et al., 2001) while the theory of feature repetition states that a stimulus priming effect accelerates responses to repeated stimuli and repeated motor responses (Mayr et al., 2003; Nieuwenhuis et al., 2006). Previous studies revealed a reduced CSE in PD (Bonnin et al., 2010; Rustamov et al., 2013) that was linked to stimulus repetition events. However, it is also reported that the CSE occurs even in the absence of feature repetitions (Duthoo et al., 2014; Tomat et al., 2020). By including a high “compatibility ratio”, i.e. higher proportion of congruent trials compared to incongruent trials (Zurawska Vel Grajewska et al., 2011) and delayed visibility of the target (Mattler, 2003), which both are known to increase flanker interference, we aimed to increase possible differences for HC and PD. We are interested in whether the CSE emerges mainly from top-down conflict monitoring or bottom-up feature repetition, which may be disentangled by evaluating response sequences, and whether patients with PD differ from HC due to impaired conflict adaptation (Botvinick et al., 1999; Rustamov et al., 2013).

We hypothesize that conflict adaptation is reduced in PD and that patients with PD show a pronounced congruency effect (Egner, 2007) in contrast to HC. Further, we hypothesize that facilitation effects do not differ among PD and HC and that activation in fronto-parietal and cingulo-opercular networks that are commonly engaged in stimulus–stimulus and stimulus–response conflicts, as it was reported in an Activation Likelihood Estimation (ALE) meta-analysis (Li et al., 2017), are reduced in conflict adaptation events in HC but not PD. Further, we expect to observe increased connectivity of conflict-processing and motor areas in PD.

## Materials and methods

### Participants

Thirty-five individuals (17 PD, 18 HC) were included in the study. All patients were seen by movement disorder specialists and met the UK Parkinson’s Disease Society Brain Bank (UKPDSBB) clinical diagnostic criteria (Hughes et al., 1992) and were on their dopaminergic medication while participating in the study. Age did not differ significantly between patients with PD and HC (p = 0.07) but gender was not balanced (PD: 14 males, 3 females; HC: 7 males, 11 females). In six patients the left side was reported to be affected most, in five cases the left side. In six cases no lateralization was reported. Table 1 summarizes demographic and clinical data of the participants.

**Table 1:** Demographic and clinic data of patients with Parkinson’s disease (PD) and healthy controls (HC)

	PD (n = 17)	HC (n = 18)
Age	58.82 (7.13)	55.22 (3.73)
Male/Female	14/3	7/11
BDI	6.18 (4.50)	3.5 (2.95)
Hoehn & Yahr stages	1.7 (range 1–3)	n.a.
UPDRS III	13.33 (7.89)	n.a.
Disease duration	5.33 (3.04)	n.a.
NMSS-PD	6.31 (5.02)	n.a.
PANDA (cognition)	22.06 (3.92)	n.a.
Levodopa	8	n.a.
MAO-inhibitor	11	n.a.
Dopamine-agonist	16	n.a.
ACE-inhibitor	3	n.a.
AChE-inhibitor	1	n.a.
Amantadine	3	n.a.

Each patient surpassed the cut-off score of 14 of the cognitive part of the Parkinson Neuropsychometric Dementia Assessment (PANDA subscale A), indicating that none of them was demented. Clinically relevant depressive symptoms were absent in both groups. However, patients with PD reached significant higher Beck’s Depression Inventory (BDI) scores compared to HC (p = 0.044). Medications are listed with the number of patients receiving them. All data (except Hoehn & Yahr stage where mean and range is depicted) are shown as mean and standard deviation (SD), age and disease as duration in years

*n.a.* not applicable, *UPDRS* Unified Parkinson's Disease Rating Scale, *BDI* Beck Depression Inventory, *NMS-PD* Non-motor Symptoms Questionnaire for Parkinson's Disease, *PANDA* Parkinson Neuropsychometric Dementia Assessment

### Experimental design and procedure

**Fig. 1:**
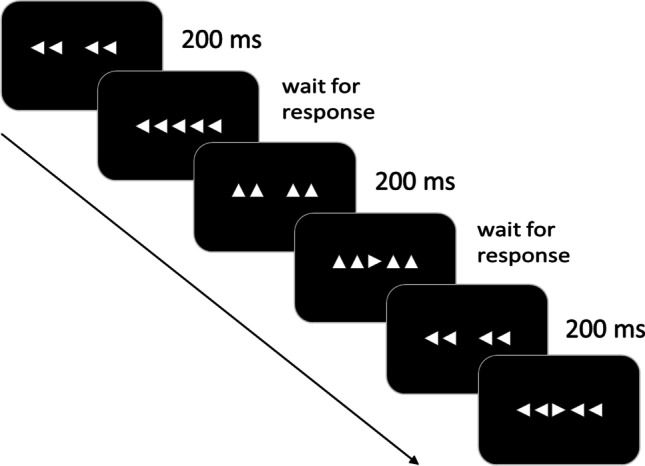
Paradigm with Flanker stimuli applied in the experiment. The stimuli were displayed on a computer screen (NordicNeuroLab 40″ 4K UHD InroomViewingDevice, NordicNeuroLab AS, Norway) that was visible to the subjects in the MRI scanner via a mirror, using Presentation software (Neurobehavioral Systems, Albany, CA). The participants were instructed to respond to the central target arrow by pressing the matching left or right key on the response device (MRI compatible response grips, Nordic neurolab) with their left or right index finger. Additionally, they were asked to respond as quickly as possible while avoiding errors

The experiment included a cognitive and health assessment to exclude participants suffering from depression or dementia that was acquired prior to the fMRI measurement. The complete investigation lasted 2 h in total. Stimuli consisted of five horizontally arranged arrows, presented on a computer screen illustrated in Fig. 1. The flanker stimulus arrays preceded the target arrow by 200 ms. The complete stimulus array remained on the screen until the participant's response was registered. The experiment contained 140 congruent events, 70 neutral events and 70 incongruent events. Presentation of the stimuli was randomized, and the interstimulus interval was jittered. A compatibility ratio with 50% congruent and 25% incongruent trials was used.

### Acquisition and statistical analysis

#### Behavioral data

Calculations were performed using SPSS 22.0 (https://www.ibm.com/software/uk/). Mean RTs to flanker events were used as outcome measures and two-tailed two sample t-tests and chi square tests in case of binary data were applied to compare demographic data between groups. Error- and post-error trials were removed from the data. Using an univariate 3 × 3 × 2 × 2 ANCOVA, a Previous trial type (C, IC, N) × Current trial type (C, IC, N) × Group (HC, PD) × Response sequence (repetition, alternation) analysis was conducted and number of errors and age were added as covariates. Post-hoc tests are Bonferroni-corrected. Homogeneity of variance was tested using the Levene-test. Data is reported as mean ± standard error of the mean (SEM) while results were considered significant with *p* < 0.05. Partial eta squared (η_P_^2^) is reported as a measure of effect size.

#### FMRI data

fMRI data was acquired using a 1.5 Tesla MR scanner (Siemens Avanto) with a 12-channel head coil. A T2*-sensitive EPI sequence was applied, 34 axial slices, no gap, matrix size of 64 × 64 mm, voxel size of 3 × 3 × 3 mm, FoV of 192 × 192 mm, TR of 2 s, TE of 30 ms, and 90° flip angle. T1-weighted structural images were acquired using a MPRAGE sequence (TR 19 ms, TE 2.91 ms, flip angle 15°, voxel size of 1.0 × 1.0 × 1.0 mm, matrix size of 256 × 256 mm). Functional images were analyzed with SPM12 (http://www.fil.ion.ucl.ac.uk/spm/). Realignment parameters were added as regressors of no interest. Results were considered relevant with family-wise-error (FWE) correction p < 0.05 for whole-brain comparison on cluster level. Activated brain areas were defined using the SPM Anatomy toolbox (Eickhoff et al., 2007).

### Psychophysiological interactions (PPI) analysis

We performed PPI analyses using the right anterior insula and left IPC as seed regions. Center coordinates of the Volumes of interest (VOIs) were defined following the results of a published ALE meta-analysis from 111 neuroimaging studies that acquired fMRI Data during conflict processing (Li et al., 2017). Spherical VOIs with a radius of 8 mm were created and Eigenvariates of the Blood Oxygen Level Dependent (BOLD) signal were extracted from the VOIs for each subject separately. PPI interaction terms were then created by convolving the extracted signal with the contrast of conflict (IC > C). The resulting PPI interaction term, BOLD signal from the VOI and task condition contrast were then added as regressors in a first-level General Linear Model (GLM), creating a main effect for the PPI-interaction term. The resulting images were then fed into a second-level two-sample t-test. Activation clusters obtained from the second-level as well as PPI results were labeled using the SPM Anatomy Toolbox (Eickhoff et al., 2007).

## Results

### Behavioral data

The preceding Levene test was significant for the Previous trial type × Current trial type × Group × Response sequence ANCOVA (F (23, 601) = 1.590, p = 0.04), therefore the dependent variable “response time” was transformed using a common logarithm. In succession, the Levene test was not significant (F (23, 601) = 0.993, p = 0.472) and all variables fulfilled the assumptions.

**Fig. 2:**
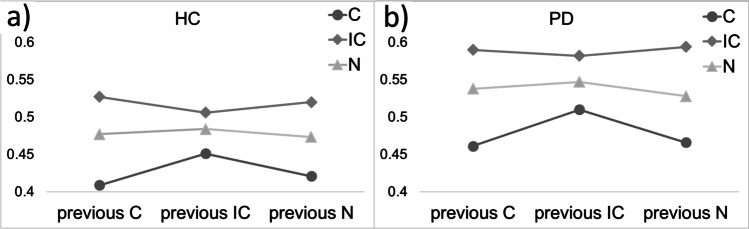
**a** Response times (in seconds) to congruent, incongruent and neutral stimuli for healthy controls (HC) as a function of previous trial type. **b** Response times (in seconds) to congruent, incongruent and neutral stimuli for patients (PD) as a function of previous trial type. Depicted data are shown as means

**Fig. 3:**
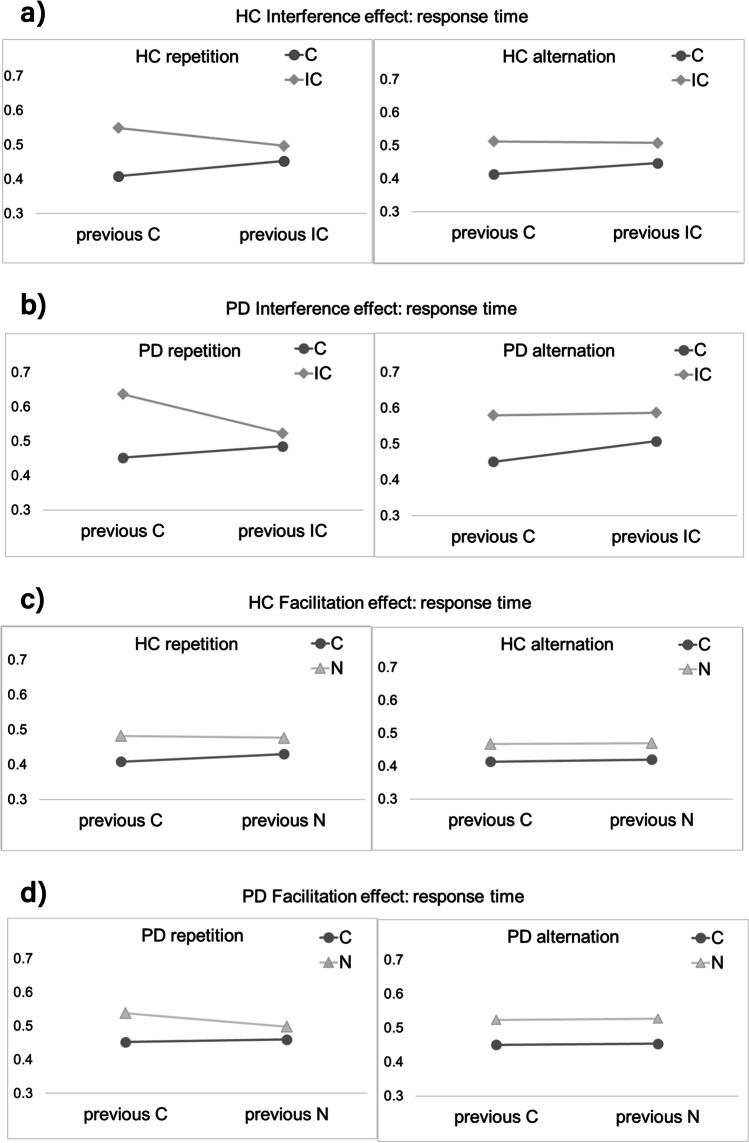
**a** Response times (in seconds) to congruent (C) and incongruent (IC) stimuli split into sequence repetition and alternation for the group of healthy controls (HC) as a function of previous trial type. **b** Response times (in seconds) to C and IC stimuli split into sequence repetition and alternation for the group of patients (PD) as a function of previous trial type. **c** Response times (in seconds) to C and neutral (N) stimuli split into sequence repetition and alternation for the group of HC as a function of previous trial type. **d** Response times (in seconds) to C and N stimuli split into sequence repetition and alternation for the group of patients (PD) as a function of previous trial type

There was a significant effect of “Current trial type” (F (2, 599) = 92.973, p < 0.001, η_P_^2^ = 0.237) and “Group” (F (1, 599) = 12.329, p < 0.001, ηP^2^ = 0.02, Fig. 2a and b) with slower RTs in PD (mean = 0.52) compared to HC (mean = 0.47). The interaction “Current trial type * Previous trial type” (F (4, 599) = 3.419, p = 0.009, η_P_^2^ = 0.022, Fig. 2a and b) and the covariates “error” (F (1, 599) = 185.571, p < 0.001, η_P_^2^ = 0.237) and “age” (F (1, 599) = 80.606, p < 0.001, ηP^2^ = 0.119) also provided significance. Bonferroni-adjusted post-hoc analysis revealed a significant difference (p < 0.001) for all pairwise comparisons regarding “Current trial type” (IC and C (0.09, 95%-CI [0.08, 0.10]), IC and N (0.04, 95%-CI [0.03, 0.05]), N and C (0.05, 95%-CI [0.04, 0.07])). RTs were faster for congruent trials (mean = 0.44) compared to incongruent trials (mean = 0.54) and neutral trials (mean = 0.50). Concerning the significant interaction “Current trial type * Previous trial type”, RTs were faster in both groups if the current and previous trial were the same trial type, regardless of the motor response alternating or repeating left or right (Fig. 3a–d, supplementary Table 2).

No significant effect appeared for “Previous trial type” (F (2, 609) = 1.624, p = 0.198, ηP^2^ = 0.005), and “Response sequence” (F (1, 609) = 2.959, p = 0.086, ηP^2^ = 0.005). There was no significant Trial type * Group interaction (Current trial type * Group (F (2, 599) = 1.367, p = 0.256, ηP^2^ = 0.005, Previous trial type * Group (F (2, 599) = 0.018, p = 0.982, ηP^2^ < 0.001, Current trial type * Previous trial type * Group (F (4, 599) = 0.016, p = 0.999, ηP^2^ < 0.001).

### FMRI data

Investigating the differential contrast of conflict adaptation (IC-IC > IC-N or C), no voxels survived in a whole-group comparison. Therefore, we decided to further investigate other aspects of conflict processing and evaluate whether we are able to replicate previous findings regarding activated brain areas during conflict processing to show that our paradigm works as expected.

**Fig. 4:**
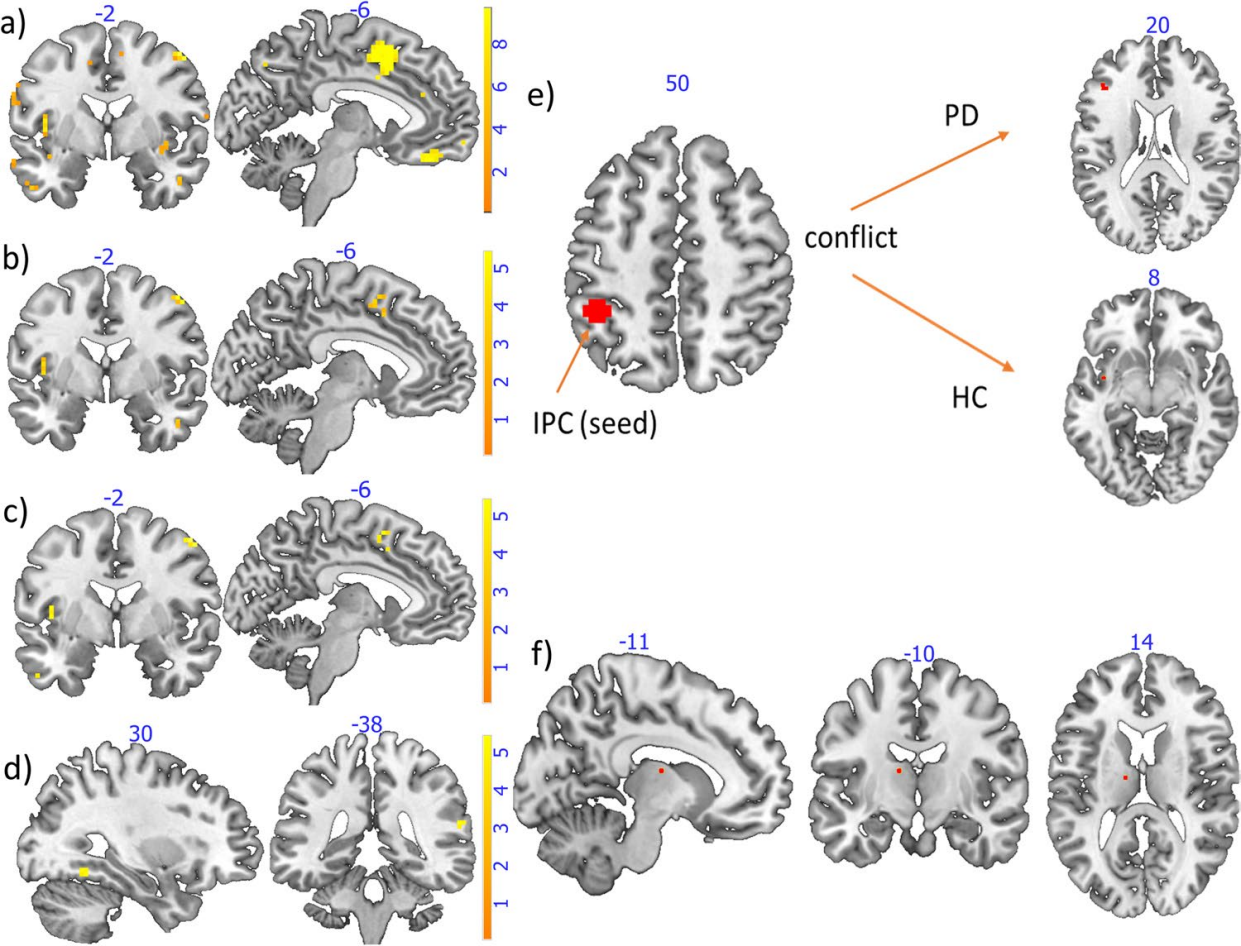
Congruency effect (both groups): **a** effect of incongruent stimuli compared to congruent and neutral stimuli IC > CN in the whole group of subjects, FWE-corrected p < 0.05, k > 50; **b** effect of incongruent stimuli compared to congruent stimuli, FWE-corrected p < 0.05, k > 50; **c** effect of incongruent stimuli compared to neutral stimuli FWE-corrected p < 0.05, k > 50; **d** group difference: HC > PD, effect of group with PD showing decreased activation: fusiform gyrus (sagittal), IPC (coronal), FWE-corrected p < 0.05, k > 1. **e** Psychophysiological interactions (PPI) with seed in IPC: changes in functional connectivity for conflict processing in contrast to non-conflict events in PD group (pars triangularis, FWE-corrected p < 0.05) and control group (insula, uncorrected); **f** PD group vs. control group; contrast: conflict (incongruent-congruent) PD > HC FWE-corrected p < 0.05, functional connectivity between the seed and this area (thalamus) is increased in the PD group compared to the control group

The analysis revealed activation of left middle temporal gyrus, left IPC and bilateral midcingulate cortex (MCC) during processing of incongruent stimuli in contrast to congruent and neutral stimuli combined (Fig. 4a, supplementary Table 1A). Contrasting incongruent to congruent stimuli (Fig. 4b, supplementary Table 1B), activated areas included left middle temporal gyrus, left IPC, left insula and left DLPFC. Contrasting incongruent to neutral stimuli (Fig. 4c, supplementary Table 1C), left middle temporal gyrus and DLPFC appeared. Comparing HC to PD, increased activation of right superior temporal gyrus (assigned to IPC), a temporal portion of the right fusiform gyrus and right middle frontal gyrus appeared in HC compared to PD (Fig. 4d), Montreal Neurological Institute (MNI)-coordinates are shown in supplementary Table 1D.

### Psychophysiological interactions (PPI) analysis

In a separate PPI analysis for the PD group with IPC as seed region, increased connectivity was revealed in left pars triangularis and left middle temporal gyrus for conflict in contrast to non-conflict processing with FWE correction p < 0.05 (Fig. 4e). No other contrast or seed revealed significant increases in connectivity with this threshold. Analyzing the HC group separately for the IPC seed and contrast of conflict compared to non-conflict processing, only with a lowered threshold increased connectivity with insular cortex was shown with p > 0.001 uncorrected (Fig. 4e). In PD compared to HC, the left IPC exhibited increased functional connectivity with the thalamus for the effect of conflict (IC > N and C). This result is significant with FWE-correction, p < 0.05 (MNI: x = 0.12, y = − 10, z = 14, 1 voxel, t = 5.78, Fig. 4f). Lowering the threshold to p = 0.001 uncorrected, this activation expands to 24 voxels.

## Discussion

A congruency sequence effect is evident in both groups (Figs. 2, 3), providing no evidence for our hypothesis that conflict adaptation is reduced in PD. This is not consistent with other studies reporting impaired sequence dependent modulation in patients with PD on medication (Fielding et al., 2005; Rustamov et al., 2013). Interestingly, effects of response sequence (motor response left or right) did not yield significance, but the interaction of current and previous trial type did. Therefore, the observed CSE can’t be explained by episodic memory retrieval (Hommel et al., 2004), substantiating that the CSE was formerly reported to be present in tasks without feature repetitions (Tomat et al., 2020), favouring conflict-induced attentional adjustment to take place. Hence, top-down conflict monitoring processes are likely the main contributors leading to the CSE in our paradigm. We anticipated a pronounced congruency effect in PD, but equivalent facilitation effects in PD. The non-significant interaction “Group * Current trial type” did not provide evidence for a larger magnitude of the congruency effect with respect to the PD group. Consequently, facilitation effects also did not differ in PD and HC. Despite a general slowing in RTs in PD that was reported previously (Adam et al., 2012), both groups showed a flanker interference effect, revealed by the significant effect of “Current trial type” and the non-significant “Group * Current trial type” interaction. This is not in line with several studies showing larger congruency effects in PD (Praamstra et al., 1998, 1999). With increasing age of the participants and increasing error rates also RTs increased in both groups, revealed by the significant effects of the covariates “Age” and “Error”.

Contrasting the HC to the PD group, fMRI analysis revealed increased activation in right IPC, DLPFC and fusiform gyrus that may be linked to superior performance in the task (Gbadeyan et al., 2016; Slotnick & White, 2013). Increased activation in middle temporal gyrus, DLPFC, IPC and insula during incongruent flanker events in contrast to congruent flanker events was observed (Fig. 4B). This is consistent with other conflict processing MRI investigations (Egner & Hirsch, 2005; Zurawska Vel Grajewska et al., 2011). DLPFC, IPC and insula are also reported to be associated with conflict processing in a domain-general pattern in a meta-analysis with ALE (Li et al., 2017). We used these regions as seeds in our PPI-analysis.

In PD, the PPI analysis revealed increased connectivity in regions reported to be involved in processes of top-down cognitive control (Egner & Hirsch, 2005). Contrasting PD against HC in the PPI analysis, increased functional connectivity of IPC and thalamus emerged in PD, which partly supports that connectivity of conflict processing and motor areas is increased in PD, because the thalamus supports motor areas in coordinating movements and is therefore also a target for deep brain stimulation in PD (Iorio-Morin & Fomenko 2020).

We observed conflict adaptation in our behavioral data for both groups, but were unable to uncover possible neural correlates for this effect. We found a significant interaction of current and previous trial type, but without an effect of repetitive or alternating response sequences, arguing against a bottom-up priming effect in our data. The results of the fMRI and PPI analyses also show predominantly top-down processes, such as activation of the DLPFC in both groups during conflict processing, with HC showing stronger activations in the group comparison. In summary we can assume that in our paradigm, the CSE emerges mainly from top-down conflict monitoring, and bottom-up feature repetition effects likely only play a minor role. In our behavioral data analysis, patients with PD differed from HC only in terms of general processing speed, and the groups did not differ in terms of congruency effects and CSE, but in our fMRI analysis, patients with PD showed reduced activation of conflict processing areas during the task compared to HC.

However, there are some limitations in our study. We did not check on differences performing with the most vs. least affected side of the body in PD prior to the experiment, and patients with PD were only tested on medication. Several studies pointed out, that medication may have a huge impact on performance in conflict tasks in PD (Djamshidian et al., 2011; Ruitenberg et al., 2019) and that dopaminergic medication may actually modulate conflict adaptation in PD (Duthoo et al., 2013). PD and HC groups also differed in terms of depressive symptoms, possibly contributing to further reducing RTs and also accuracy in our PD group (Herzallah et al., 2017). Previous research reported a lacking CSE in PD (van Wouwe et al., 2014) and basal ganglia dysfunction facilitates initiation of movement following irrelevant external cues (Praamstra et al., 1998, 1999). However, inhibition of irrelevant flankers is likely modulated with medication in PD (Duthoo et al., 2013) and the CSE may also be rather linked to amplification of task-relevant information instead of inhibition of task-irrelevant information (Egner & Hirsch, 2005). Future research should focus on controlling medication status in PD and attempting to further improve probe task methodology to achieve a better understanding of possible impairments of conflict-induced adaptation of cognitive control in PD.

## Conclusions

We confirmed equivalent facilitation effects in PD and HC and patients with PD differed from HC only in terms of general processing speed. No evidence was provided for a pronounced congruency effect or for reduced conflict adaptation in PD. The CSE probably emerges mainly from top-down conflict monitoring in our paradigm. In our fMRI analysis, we were not able to provide evidence that fronto-parietal and cingulo-opercular networks show reduced activation during conflict adaptation in PD, but we revealed that connectivity of conflict processing and motor-related areas is increased in PD.

## Supplementary Information

Below is the link to the electronic supplementary material.

Supplementary file1 (DOCX 20 kb)

## Data Availability

The datasets generated and analyzed during the current study are available from the corresponding author, without undue reservation, on reasonable request.
