# Supplementary material for: Conflict adaptation and related neuronal processing in Parkinson’s disease
Source: Brain Imaging Behav. 2021 Aug 27;16(1):455–63. doi: 10.1007/s11682-021-00520-w (PMC8825575; doi:10.1007/s11682-021-00520-w)
Supplement: Supplementary file 1 — Supplementary file1 (DOCX 20 kb) [file 11682_2021_520_MOESM1_ESM.docx]

SUPPLEMENTARY MATERIAL

Conflict adaptation and related neuronal processing in Parkinson’s disease

Rea Rodriguez-Raecke^1,2^, Christoph Schrader^2^, Pawel Tacik^2,4^, Dirk Dressler^2^, Heinrich Lanfermann^3^ & Matthias Wittfoth^3^

^1 Diagnostic and Interventional Neuroradiology, University Hospital, RWTH Aachen University, Germany^

^2 Department of Neurology, Hannover Medical School, Hannover, Germany^

^3 Department of Neuroradiology, Hannover Medical School, Hannover, Germany^

^4 Department of Neurodegenerative Diseases and Geriatric Psychiatry, University of Bonn Medical Center, Bonn, Germany^

*Corresponding author and person to whom reprint requests should be addressed:*

Rea Rodriguez-Raecke, PhD, ORCID-ID: 000-0001-89809698

Diagnostic and Interventional Neuroradiology

Uniklinik RWTH Aachen

Pauwelsstraße 30

D-52074 Aachen, Germany

Tel: +49 (0)241-80-37438

Fax: +49 (0)241 80-82155

*Email*: [rrodriguez@ukaachen.de](mailto:rrodriguez@ukaachen.de)

***Keywords:*** *fMRI, congruency sequence effect, Parkinson’s disease, cognitive control, conflict adaptation*

*Table 1: MNI-coordinates for the Effect of congruency IC > CN, IC > C; IC > N and the effect of group*

| **MNI coordinates A) Congruency effect IC>NC, FWE < 0.05, k > 50** | | | | | | | | | | | | |  |  |  |
| --- | --- | --- | --- | --- | --- | --- | --- | --- | --- | --- | --- | --- | --- | --- | --- |
| **x** | | **y** | | **z** | | | **voxels** | | **t** | | **Brain region** | | |  |  |
| **-54** | | **-67** | | **-1** | | **6369** | | | **9.61** | | **L Middle Temporal Gyrus (hOc4Ia)** | | | | |
| **-51** | | **-64** | | **-13** | | **6369** | | | **9.54** | | **L Inferior Occipital Gyrus** | | | | |
| **-48** | | **-28** | | **47** | | **6369** | | | **9.43** | | **L Inferior Parietal Cortex (IPC)** | | | | |
| **66** | | **-40** | | **23** | | **231** | | | **8.29** | | **R Inferior Parietal Cortex (IPC, area 40)** | | | | |
| **63** | | **-19** | | **44** | | **231** | | | **8.13** | | **R Postcentral Gyrus** | | | | |
| **63** | | **-28** | | **44** | | **231** | | | **7.71** | | **R SupraMarginal Gyrus** | | | | |
| **45** | | **-82** | | **17** | | **885** | | | **8.26** | | **R Middle Occipital Gyrus** | | | | |
| **48** | | **-82** | | **-1** | | **885** | | | **7.97** | | **R Inferior Occipital Gyrus** | | | | |
| **48** | | **-76** | | **-10** | | **885** | | | **7.64** | | **R Inferior Occipital Gyrus** | | | | |
| **9** | | **-31** | | **47** | | **53** | | | **6.39** | | **R Midcingulate Cortex (MCC)** | | |  | |
| **-9** | | **-22** | | **47** | | **53** | | | **5.96** | | **L Midcingulate Cortex (MCC)** | | |  | |
| **0** | | **-31** | | **44** | | **53** | | | **5.5** | | **L Midcingulate Cortex (MCC)** | | |  | |
|  | | | | | |  |  |  |  |  |  |  |  |  |  |
| **MNI coordinates B) Congruency effect IC > C, FWE < 0.05, k > 50** | | | | | | | | | | | | |  |  |  |
| **x** | | **y** | | **z** | | | **voxels** | | **t** | | **Brain region** | | |  |  |
| **-54** | | **-67** | | **-1** | | **317** | | | **7.31** | | **L Middle Temporal Gyrus (hOc4Ia)** | | |  |  |
| **-51** | | **-64** | | **-13** | | **317** | | | **7.26** | | **L Inferior Occipital Gyrus** | | |  |  |
| **-66** | | **-13** | | **-1** | | **317** | | | **6.83** | | **L Middle Temporal Gyrus** | | |  |  |
| **-48** | | **-28** | | **47** | | **79** | | | **7.19** | | **L Inferior Parietal Cortex (IPC)** | | |  |  |
| **-60** | | **-22** | | **44** | | **79** | | | **5.71** | | **L Inferior Parietal Cortex (IPC)** | | |  |  |
| **-60** | | **-31** | | **44** | | **79** | | | **5.42** | | **L Inferior Parietal Cortex (IPC)** | | |  |  |
| **-57** | | **11** | | **-13** | | **52** | | | **6.61** | | **L Temporal Pole** | | |  |  |
| **-54** | | **17** | | **-4** | | **52** | | | **6.27** | | **Area 45** | |  |  |  |
| **-45** | | **5** | | **-1** | | **52** | | | **5.76** | | **L Insula Lobe** | | |  |  |
| **-60** | | **5** | | **29** | | **56** | | | **5.98** | | **L Precentral Gyrus** | | |  |  |
| **-60** | | **17** | | **17** | | **56** | | | **5.78** | | **L IFG (p. Opercularis)** | | |  |  |
| **-42** | | **5** | | **35** | | **56** | | | **5.25** | | **L Precentral Gyrus** | | |  |  |
| **-33** | | **62** | | **2** | | **95** | | | **5.9** | | **L Superior Frontal Gyrus (DLPFC)** | | |  |  |
| **-42** | | **44** | | **26** | | **95** | | | **5.85** | | **L Middle Frontal Gyrus (DLPFC)** | | |  |  |
| **-39** | | **56** | | **14** | | **95** | | | **5.72** | | **L Middle Frontal Gyrus (DLPFC)** | | |  |  |
| **51** | | **-40** | | **-22** | | **95** | | | **5.01** | | **R Inferior Temporal Gyrus** | | |  |  |

| **MNI coordinates C) Congruency effect IC > N, FWE < 0.05, k > 50** | | | | | | | | | | |  | | |
| --- | --- | --- | --- | --- | --- | --- | --- | --- | --- | --- | --- | --- | --- |
| **x** | | **y** | | **z** | | **voxels** | | **t** | | **Brain region** | | | |
| **-54** | **-67** | | **-1** | | **123** | | **6.83** | | **L Middle Temporal Gyrus** | | | |  |
| **-51** | **-64** | | **-13** | | **123** | | **6.79** | | **L Inferior Occipital Gyrus** | | | |  |
| **-48** | **-79** | | **-7** | | **123** | | **6.24** | | **Area hOc4la** | | |  |  |
| **-33** | **62** | | **5** | | **79** | | **5.73** | | **Area Fp1** | | | |  |
| **-33** | **47** | | **35** | | **79** | | **5.68** | | **L Middle Frontal Gyrus (DLPFC)** | | | |  |
| **-36** | **59** | | **14** | | **79** | | **5.64** | | **L Middle Frontal Gyrus (DLPFC)** | | | |  |

| **MNI coordinates D) Effect of group HC > PD, FWE < 0.05, k > 1** | | | | | |  |
| --- | --- | --- | --- | --- | --- | --- |
| **x** | **y** | **z** | **voxels** | **t** | **Brain region** | |
| **60** | **-37** | **20** | **3** | **5.42** | **R Superior Temporal Gyrus (IPC)** | |
| **30** | **-52** | **-13** | **4** | **5.27** | **R Fusiform Gyrus (area FG3)** | |
| **36** | **50** | **29** | **2** | **4.81** | **R Middle Frontal Gyrus (DLPFC)** | |

*Table 2: Mean response times regarding the interaction of current trial type and previous trial type combined for both groups.*

| **Current trial type** | **Previous trial type** | **Mean**  **RT** | **Standard error** | **95%-**  **confidence interval** | |
| --- | --- | --- | --- | --- | --- |
|  |  |  |  | **Lower bound** | **Upper bound** |
| **congruent** | **congruent** | **0.430** | **0.006** | **0.419** | **0.441** |
|  | **incongruent** | **0.473** | **0.011** | **0.452** | **0.495** |
|  | **neutral** | **0.439** | **0.011** | **0.417** | **0.461** |
| **incongruent** | **congruent** | **0.566** | **0.011** | **0.544** | **0.588** |
|  | **incongruent** | **0.536** | **0.006** | **0.525** | **0.547** |
|  | **neutral** | **0.552** | **0.011** | **0.53** | **0.573** |
| **neutral** | **congruent** | **0.502** | **0.011** | **0.48** | **0.523** |
|  | **incongruent** | **0.509** | **0.011** | **0.487** | **0.53** |
|  | **neutral** | **0.493** | **0.006** | **0.482** | **0.504** |
